# Green-synthesized manganese nanoparticles for antibiotic and heavy metal remediation: integrated experimental and computational evaluation

**DOI:** 10.7717/peerj.21443

**Published:** 2026-07-09

**Authors:** Jumana Ghannam, Danah Aloumi, Sahar S. Alghamdi, Afrah E. Mohammed

**Affiliations:** 1 Department of Biology, College of Science, Princess Nourah bint Abdulrahman University, Riyadh, Saudi Arabia; 2 Department of Pharmaceutical Sciences, College of Pharmacy, King Saud bin Abdulaziz University for Health Sciences, Riyadh, Saudi Arabia; 3 King Abdullah International Medical Research Center, Riyadh, Saudi Arabia; 4 Pharmaceutical Care Department, King Abdulaziz Medical City, National Guard Health Affairs, Riyadh, Saudi Arabia; 5 Microbiology and Immunology Unit, Natural and Health Sciences Research Center, Princess Nourah bint Abdulrahman University, Riyadh, Saudi Arabia

**Keywords:** Manganese nanoparticles, Heavy metals, Nanotechnology, Tylosin, Soil remediation

## Abstract

The current study investigates the use of *Acacia tortilis* seed powder to synthesize innovative manganese nanoparticles (MnNPs) through an eco-friendly process providing an eco-friendly alternative to conventional chemical methods for remediating co-contaminated soils. These MnNPs are designed to remediate soils contaminated with tylosin (TYL) antibiotics and heavy metals (HMs), including lead, cadmium, and copper. The synthesized MnNPs were characterized using UV-visible spectrophotometry (UV-Vis), scanning electron microscopy (SEM), transmission electron microscopy (TEM), Fourier-transform infrared spectroscopy (FTIR), dynamic light scattering (DLS), and zeta potential analysis, confirming successful formation with an average size of 84.6 nm and a surface charge of −32.09 mV, indicating good stability. Remediation experiments demonstrated significant removal efficiencies, achieving 39.8% reduction of TYL and 51.19% reduction of cadmium (Cd) in industrial soil, compared to lower efficiencies in stable soil (16.1% and 14.9%, respectively), highlighting the influence of soil properties on treatment performance. Compared to conventional remediation approaches, MnNPs offer a cost-effective and environmentally sustainable alternative, although their efficiency varies depending on soil composition and contaminant interactions. In addition, *in silico* analysis it revealed that certain phytochemicals, such as 4-ethenyl-2,6-dimethoxyphenol, exhibit rapid degradation (half-life = 16 days), supporting their suitability for environmental applications. This study addresses the limited integration of green nanoparticle synthesis with combined experimental and computational environmental assessment. However, further research is required to evaluate the long-term stability, ecological risks, and large-scale applicability of MnNPs in diverse soil systems.

## Introduction

Urbanization and agricultural activities contribute significantly to environmental and human health challenges, primarily through soil contamination with organic and inorganic pollutants (Sathish et al., 2024). Veterinary antibiotics (VAs), extensively used in livestock farming for disease prevention and growth enhancement, pose notable environmental and public health risks (Joseph, Moturi Wilkister & Ogendi, 2024). Tylosin (TYL), a macrolide antibiotic, is used both for treatment of infectious diseases and as a growth promoter (Barshevskaya et al., 2024). Once administered, TYL is only partially metabolized in animals, leaving residues in manure that can contaminate farmland soil (Hu & Coats, 2009). Additionally, TYL can enter the soil through wastewater irrigation, binding with soil particles and potentially moving to surface water or groundwater, thus becoming available for plant uptake (Youssef et al., 2020). Residual antibiotics in the environment can suppress microbial growth, harm algae and plants, and promote antibiotic resistance (Albarano et al., 2024). The uptake of antibiotics and resistance genes through food chains further threatens human health and reduces the efficacy of bacterial infection treatments (Ifedinezi et al., 2024). Microbial and enzymatic degradation represents one of the primary natural attenuation pathways for antibiotic residues in livestock manure. Recent reviews highlight that microorganisms and their extracellular enzymessuch as *β*-lactamases, esterases, laccases, and peroxidases facilitate antibiotic breakdown through hydrolysis, oxidation, and reduction reactions, thereby reducing antibiotic persistence, antimicrobial resistance (AMR) selective pressure, and the abundance of antibiotic resistance genes (ARGs) in manure systems (Zhao et al., 2025). Engineered manure-management processes, including aerobic composting and anaerobic digestion, further enhance degradation by promoting high-temperature inactivation, suppressing horizontal gene transfer, and optimizing microbial activity (Zubair et al., 2023).

Despite these advantages, biological degradation often remains incomplete, leading to the formation and accumulation of intermediate transformation products that may persist in soil or exhibit greater toxicity than the parent compounds (Gao et al., 2025). Moreover, the efficiency of microbial and enzymatic pathways is highly sensitive to environmental variables such as pH, temperature, oxygen availability, and microbial community composition, which limits their reliability in complex or co-contaminated soils. These constraints underscore the need for complementary remediation strategies capable of enhancing degradation efficiency while minimizing harmful byproducts. In this context, nanomaterial-assisted approaches particularly metal-based nanoparticles offer promising advantages due to their high reactivity, catalytic properties, and ability to facilitate both adsorption and degradation processes

Regions heavily reliant on manure for agriculture, such as Al-Kharj City in Riyadh province, Saudi Arabia, show high concentrations of VAs. A study in this region detected significant mean concentrations of tetracycline (TC), doxycycline (DC), oxytetracycline (OTC), and sulfamethoxazole (SMZ) (Al-Wabel et al., 2021). Thus, there is a crucial need for efficient and cost-effective technologies to remediate antibiotic residues in the soil.

Heavy metals (HMs), characterized by high atomic weights and densities, also pose increasing threats to human health, food safety, and soil ecosystems due to industrial growth (Rabha & Dhaneesh, 2024). Industrial emissions spread through air, water, and soil, while sewage wastewater used in irrigation can further contaminate soil (Abdullahi et al., 2024). Heavy metals such as cadmium (Cd), copper (Cu), and lead (Pb) are particularly concerning. Cadmium (Cd) is toxic and accumulates in crops, posing severe health risks through crop accumulation (Yang et al., 2024). While copper (Cu) is essential for plants and humans, excess concentration disrupts plant growth and soil health, causing organ damage and neurological issues in humans. Lead (Pb) reduces soil fertility and microbial diversity, while in humans, it damages the nervous system and causes anemia (Jahandari & Abbasnejad, 2024). A study in an industrial area south of Riyadh found heavy metal concentrations in soil, highlighting the need for immediate action to mitigate these risks (Aloud et al., 2022).

Various remediation strategies for soil contaminated hazardous substances include physical, chemical, and biological methods (Kumar et al., 2023). Physical methods, like excavation, are costly and can cause secondary issues (Awa & Hadibarata, 2020). Chemical methods, while effective, may introduce additional pollutants (Blenis et al., 2023). Bioremediation is less harmful but sensitive to environmental factors (Dhaliwal et al., 2020).

Nanotechnology, focusing on materials sized between 1–100 nanometers, offers promising solutions due to the unique properties of nanoparticles (NPs), such as a high surface area-to-volume ratio (Egesoy et al., 2024). The green synthesis of NPs using plant extracts is gaining attention for being eco-friendly, non-toxic, and cost-effective. Plant extracts act as reducing agents, aiding in converting metal ions into metal NPs, providing an advantageous one-step process without pathogenic risks (Osman et al., 2024). Metallic nanoparticles (MNPs) are especially suited for environmental remediation, reacting well with both organic pollutants and heavy metals (Kaur et al., 2022). A recent study investigated the potential of integrating anaerobic dechlorinating bacteria (such as Dehalobacterium and Dehalogenimonas) with nanoscale zero-valent iron (nZVI) for the detoxification of organic contaminant. However, the findings showed that nZVI effectively transformed chloroform into dichloromethane, which was subsequently mineralized by Dehalobacterium into non-toxic end products. Although soluble compounds released from nZVI were found to inhibit bacterial activity, this inhibitory effect is likely to be reduced under field conditions due to dilution in the subsurface environment (Wang et al., 2024). Recent advances in nano-bioremediation highlight synergies between nanoparticles and microorganisms for heavy metal removal, where NPs enhance microbial activity, immobilization, and degradation processes in contaminated soils, overcoming limitations of standalone biological methods (Kamyab et al., 2025).

Manganese nanoparticles (MnNPs) have proven effective due to their catalytic properties and significant surface area (Sobańska et al., 2021). With applications in antimicrobial activities, and pollutant degradation (Zhang et al., 2022), MnNPs have shown great potential.

Recent studies further highlight the growing integration of biological systems with nanomaterials for enhanced remediation performance. For example, bacteria-supported manganese oxide (BMOx) synthesized *in situ* by the heavy-metal-resistant bacterium *Ralstonia pickettii* demonstrated strong catalytic activity, activating peroxymonosulfate (PMS) and achieving 97.6% ciprofloxacin degradation within 15 min under neutral pH conditions (Zhu et al., 2025). This illustrates how microbial-nanomaterial hybrids can accelerate antibiotic breakdown through synergistic redox processes. Similarly, bio-mediated iron-doped manganese oxide nanoparticles (MnO_2_:Fe^3^^+^) produced using *Tridax procumbens* extract achieved 94.23% degradation of tetracycline hydrochloride under visible light, demonstrating the potential of plant-assisted nanomaterials as low-cost photocatalysts for antibiotic removal (Majani et al., 2025). In another example, green-synthesised Mn–Mg binary oxide nanoparticles derived from *Ficus exasperata* exhibited high crystallinity, uniform nanoscale size (∼92 nm), and a large surface area, enabling effective immobilization of multiple heavy metals (zinc (Zn), nickel (Ni), iron (Fe), Cu, chromium (Cr)) in contaminated soils (Oluchi et al., 2025). Together, these studies underscore the emergence of bio-nanomaterial remediation frameworks, where microorganisms, fungi, and plant metabolites act as reducing, stabilizing, or catalytic partners that enhance nanoparticle performance and environmental compatibility.

*Acacia tortilis*, a drought-resistant halophyte native to regions such as Ethiopia, Yemen, Sudan, Somalia, Palestine, Kenya, Tanzania, and Saudi Arabia, is rich in phytochemicals such as flavonoids, alkaloids, saponins, cardiac glycosides, and catechic tannins (Snoussi, Najett & Djamel, 2020). This study investigates the potential of *Acacia tortilis* subsp. spirocarpa seed extract for the green synthesis of environmentally beneficial manganese nanoparticles (MnNPs) and their application in soil remediation. A range of techniques was used to analyze the characteristics of the synthesized MnNPs. These techniques include UV-visible spectrophotometry (UV-Vis), dynamic light scattering (DLS), scanning electron microscopy (SEM), transmission electron microscopy (TEM), and Fourier-transform infrared spectroscopy (FTIR). Thereafter, the remediation potential of the synthesized MnNPs has been assessed in mitigating Tylosin (TYL) and heavy metals (HMs) from two different soil types. The key chemical compounds in *A. tortilis* seed extract were identified using gas chromatography–mass spectrometry (GC–MS) and their eco-toxicological and environmental behavior through *in silico* analyses were assessed. By combining experimental and computational approaches, this research seeks to provide insights into the potential of MnNPs for mitigating soil contamination by antibiotics and heavy metals while addressing the environmental risks associated with their application. The novelty of this study lies in integrating green synthesis of MnNPs from *A. tortilis* seeds with both experimental remediation assessment and computational environmental risk evaluation, providing a comprehensive approach for addressing soil contamination. Therefore, this study is based on the following hypotheses:

(i) Green-synthesized MnNPs can effectively reduce antibiotic and heavy metal concentrations in co-contaminated soils.

(ii) Remediation efficiency varies depending on soil type and physicochemical properties.

(iii) Phytochemical components involved in nanoparticle synthesis influence degradation pathways and environmental fate.

### Methodology

#### Chemicals and reagents

Manganese sulfate (MnSO_4_), lead chloride (PbCl_2_), cupric chloride (CuCl_2_), and cadmium sulfate (CdSO_4_), were sourced from the Laboratory of Princess Nourah bint Abdulrahman University in Riyadh, Saudi Arabia. Methanol (CH_3_OH) and sodium chloride (NaCl) were obtained from Microbiology and Immunology Unit, Natural and Health Sciences Research Center, Princess Nourah bint Abdulrahman University, Riyadh, Saudi Arabia. Acetonitrile were obtained from Abdullatif H. Abuljadayel & Sons company. *Acacia tortilis* var. ssp. spirocarpa seeds were obtained from the Seed Bank of the Royal Commission of Riyadh City, Riyadh, Saudi Arabia. Tylosin tartrate was obtained from the Arab Company for Medical Products. Distilled water used in the laboratory was prepared using Thermo Scientific. GenPure Pro UV/UF type (Germany).

### Biosynthesis of manganese nanoparticles (MnNPs)

**Figure 1:**
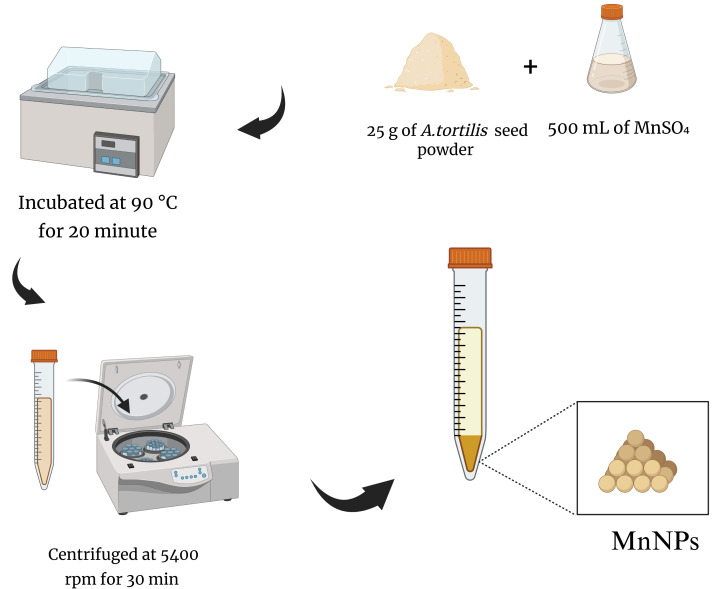
The steps of MnNPs synthesis. The steps of MnNPs synthesis using *A. tortilis* seed powder.

To begin, impurities were removed from *A. tortilis* seeds. The seeds were then dried in an oven at 70 °C for 24 h to eliminate moisture. After drying, the seeds were ground into a fine powder. In clean Erlenmeyer flasks, 25 g of the *A. tortilis* seed powder was placed, and 500 mL of freshly prepared one mM manganese (II) sulfate (MnSO_4_) solution was added. The mixture was gently stirred by hand and incubated at 90 °C with agitation at 44 rpm for 20 min. After incubation, the solution was filtered and transferred into 15 mL conical centrifuge tubes. Centrifugation was performed at 5,400 rpm for 30 min to remove the supernatant. The resultant pellets were washed several times with distilled water to isolate pure MnNPs. These pellets were then dried at room temperature for 24 h and stored for further study (Fig. 1).

### Characterization of MnNPs and *A. tortilis* phytochemicals

The properties of the synthesized MnNPs were determined using various techniques. Ultraviolet-visible (UV-Vis) spectral analysis was conducted with an Evolution 201 UV-Visible spectrophotometer (Thermo Fisher Scientific, Waltham, MA, USA). The reaction mixture was tested within a wavelength range of 200 to 450 nm after 24 h, with distilled water acting as a blank. For dynamic light scattering (DLS) and zeta potential measurements, a Zetasizer (NANO ZSP; Malvern Instruments Ltd.) was used to analyze the hydrodynamic size distribution and polydispersity index (PDI). Scanning electron microscopy (SEM) and transmission electron microscopy (TEM) were employed to analyze the biosynthesized nanoparticles. SEM (JEOL, Tokyo, Japan), equipped with energy-dispersive X-ray (EDX) capabilities, investigated the surface morphology and elemental composition of the nanoparticles. Meanwhile, TEM (JEOL, Tokyo, Japan) evaluated the size and detailed morphology of the nanoparticles. Fourier-transform infrared (FTIR) spectroscopy (SPECTRUM100; Perkin-Elmer, Waltham, MA, USA) analyzed the functional groups in the phytoconstituents responsible for reducing and capping the nanoparticles. The scanning range for FTIR was set between 4,000–1,000 cm^−^^1^. Gas chromatography–mass spectrometry (GC–MS) (Agilent Technologies 7890B Gas Detector and Agilent Technologies 7000D Mass Spectrometer Detector; Agilent Technologies, Santa Clara, CA, USA) was selected as the method of chemical analysis to identify the active metabolites in the methanolic seed extract of the *A. tortilis*.

### Soil experiments

#### Soil sampling

Soil samples were collected from two distinct regions in Saudi Arabia. The first sample (Si) was obtained from an industrial area in Riyadh (24°33′04.1″N, 46°52′48.4″E), while the second sample (SS) was collected from a private horse stable in the Medina region (24°39′09.0″N, 39°32′28.4″E). The soil samples were taken from a depth of 0–5 cm and processed by passing through a two mm sieve, thoroughly washing with distilled water, and air-drying in an oven at 70 °C for 24 h. Once dried, the samples were stored in plastic bags for subsequent use in pot experiments.

### Soil characterization

Soil characterization involved measuring pH levels and electrical conductivity (Estefan, Sommer & Ryan, 2013). The total organic carbon (TOC) and organic matter (OM) content were determined (Walkley & Black, 1934), and total nitrogen (N) content was assessed (Nelson & Sommers, 1972). The presence of calcium carbonate (CaCO_3_) was also tested (Horváth, Opara-Nadi & Beese, 2005), and soil texture analysis was conducted (Gee & Or, 2018). These tests provided a comprehensive overview of the soil’s chemical and physical properties.

### Soil treatment with tylosin (TYL)

A total of 10 grams of clean soil from the industrial area (Si) and the horse stable (SS) were placed on clean plates, each in triplicate. 10 mL of TYL solution was then added to 10 g of Si and SS soil. The plates containing the treated soil were left to dry at room temperature. After drying, the soil was thoroughly mixed to ensure homogenization.

### Soil treatment with heavy metals

Five grams of clean soil from both the industrial area (Si) and the horse stable (SS) were placed on clean plates. Each soil type was spiked with solutions of Cd, Pb, and Cu at a concentration of 10 ppm. Specifically, five mL of each heavy metal solution was added to the five grams of clean soil. The treated soils were then dried at room temperature to remove moisture before thoroughly mixing to ensure homogenization.

### Soil treatments with MnNPs

One gram of each type of soil contaminated with Tylosin (TYL) was transferred into 50 mL Falcon tubes. To prepare for the treatment, 40 mg of MnNPs were dissolved in one mL of distilled water within 1.5 mL Eppendorf tubes and sonicated for 25 min at room temperature. The contaminated soil samples were then treated with the nanomaterial solution, while the control group received only one mL of distilled water. All samples were placed on a shaker and agitated for 24 h at 150 rpm at room temperature then subjected to TYL concentration measurements. For heavy metal remediation, half a gram of soil contaminated with Cd, Pb, and Cu was placed in individual 50 mL Falcon tubes. Each soil sample was treated with 50 mg of MnNPs, previously dissolved in one mL of distilled water and sonicated for 25 min at room temperature. This MnNPs solution was then added to the Falcon tubes containing the contaminated soil. A control group was treated with only 1 mL of distilled water. The samples were agitated on a shaker for 24 h at 150 rpm at room temperature then subjected to Cd, Pb and Cu measurements. All experimental steps were conducted in triplicate. The experimental design was adapted from previously reported soil microcosm studies evaluating nanomaterial-based remediation of contaminated soils, with slight modifications (Samani et al., 2024).

### Soil sample preparation for tylosin detection using liquid chromatography–mass spectrometry

After 24 h of shaking, the soil samples in Falcon tubes were treated with 0.8 g of sodium chloride (NaCl) and homogenized using a vortex mixer at 2,800 rpm for 1 min. Each sample was then treated with 8.0 mL of acetonitrile and vortexed at the same speed for an additional 5 min. The homogenized samples were stored in a freezer at −20 °C for over 16 h (overnight). Following freezing, a two mL aliquot of the supernatant was separated and centrifuged at 5,410 rpm for 10 min using Thermo Scientific microcentrifuge tubes. After centrifugation, one mL of the supernatant was extracted and transferred to vials for further analysis. All extraction and analysis steps were performed in triplicate. The concentration of Tylosin in the soil was analyzed using an liquid chromatography–mass spectrometry (LC–S)/MS system, consisting of an Agilent 1290 Series LC system combined with a Triple Quad LC-MS 6460 detector. Sample separation was conducted on an Agilent Zorbax Eclipse Plus C18 RRHD column (2.1 × 50 mm, 1.8-micron particle size), using a mobile phase of 50% acetonitrile/formic acid (0.1%) and 50% water/formic acid (0.1%) at a flow rate of 0.2 mL per minute, with the oven temperature set to 35 °C. A 1.0-μL sample was injected for analysis. This methodology followed the protocols established by Paranhos et al. (2021).

### Soil sample preparation for heavy metals analysis by inductively coupled plasma optical emission spectrometry

Inductively coupled plasma optical emission spectrometry (ICP-OES) was utilized to determine concentrations of Cd, Cu, and Pb in soil samples, both before and after treatment with MnNPs. The ICP-PRO (Thermo Fisher Scientific, Serial No. iCAP PRO10143) was used in conjunction with a microwave digestion system (Multiwave GO Plus; Anton Paar). Air-dried soil samples were passed through an appropriate sieve to remove unwanted particles. Subsequently, 0.5 g of each sample was digested using the Anton Paar Microwave Digestion System in a mixture of nitric and hydrochloric acids (3:1 ratio). The digested samples were filtered and diluted as necessary. Heavy metals analysis was conducted at the GECO (Geotechnical & Environmental Company Ltd) laboratories.

The removal efficacy of antibiotics and heavy metals was calculated by using the following formula:

\begin{eqnarray*}\text{Removal Efficacy}~ \left( \% \right) = \left( \frac{\text{Initial Concentration}-\text{Final Concentration}}{\text{Initial Concentration}} \right) \times 100. \end{eqnarray*}

### Statistical analysis

All experiments were conducted in triplicate, and results are presented as mean ± standard deviation. Statistical analyses were performed using GraphPad Prism. Differences between treatment groups were evaluated using one-way ANOVA followed by Tukey’s post-hoc test to determine pairwise significance. The significance level of *p* < 0.05 was considered statistically significant.

### Computational eco-toxicological and environmental fate assessment of *Acacia tortilis* seed compounds for MnNPs applications

For EcoToxicity, *Earthworm Toxicity Model (CONCERT v. 1.0.0)* were applied to predict acute terrestrial toxicity (Lavado et al., 2022). Additionally, to evaluate the compounds’ environmental behavior, the *Persistence (soil) quantitative model (IRFMN v. 1.0.1)* was employed. This model predicted the stability and degradation of the compounds in soil environments, providing a deeper understanding of their environmental fate and distribution (Benfenati et al., 2019). The computational analyses were designed to complement the experimental findings, offering predictive insights into the potential risks and remediation capabilities of the MnNPs synthesized from *A. tortilis*.

**Figure 2:**
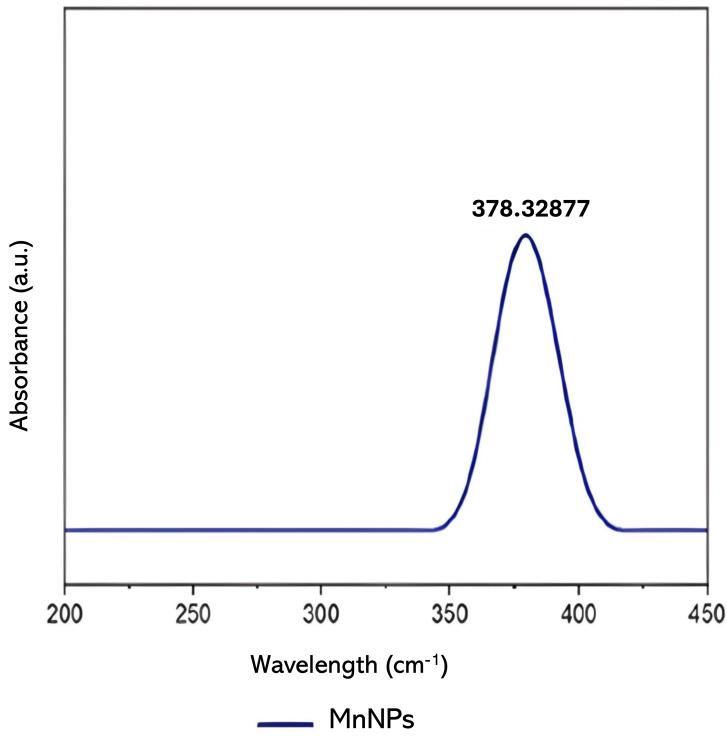
The UV-VIS spectra of MnNPs. The UV-VIS spectra reveal absorbance peaks of MnNPs synthesized using *A. tortilis* seed powder, confirming their successful formation.

## Results

### Ultraviolet-visible spectroscopy

The UV-visible spectroscopy analysis of the MnNPs, presented in Fig. 2, reveals absorbance peaks in the wavelength range of 200–450 nm, with a prominent peak at 378.32 nm. Confirming the successful synthesis of MnNPs using *A. tortilis* seed powder.

### Dynamic light scattering (DLS) and zeta potential (ZP)

**Figure 3:**
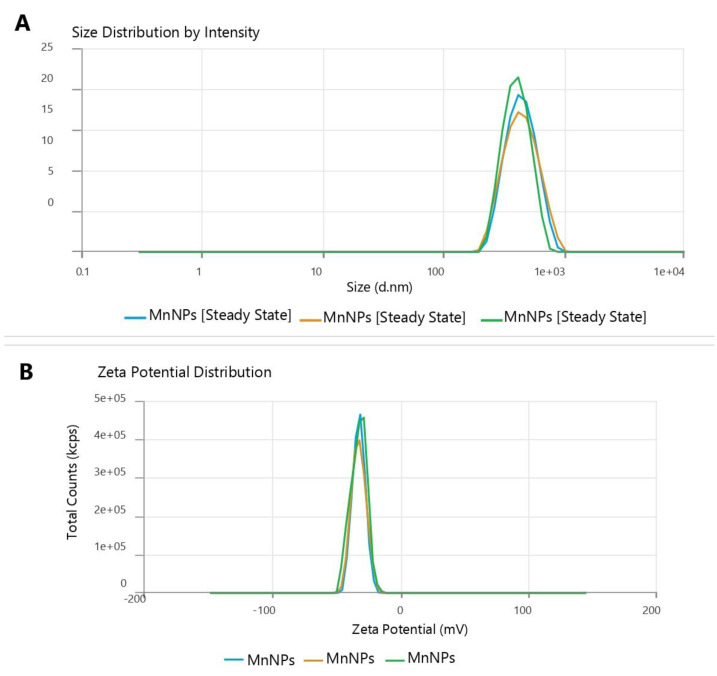
Size distribution and zeta potential distribution for the MnNPs. (A) Size distribution and (B) zeta potential distribution for the MnNPs fabricated by *A. tortilis* seed powder.

The synthesized manganese nanoparticles (MnNPs) exhibited an average hydrodynamic size of 404.1 nm with a polydispersity index (PDI) of 0.14, as shown in Fig. 3A. The zeta potential (ZP) was measured at −32.09 mV, as illustrated in Fig. 3B.

### Fourier-transform infrared spectroscopy

**Figure 4:**
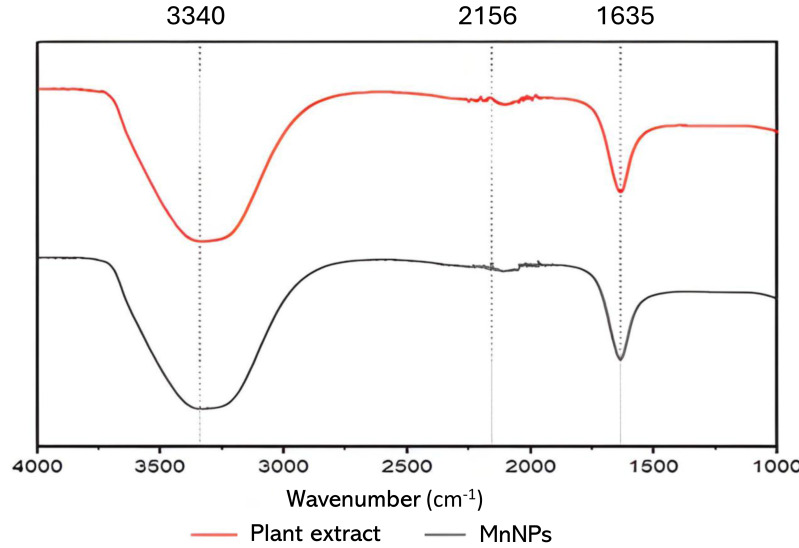
The FTIR analysis of *A. tortilis* plant extract and MnNPs. The FTIR spectra of *A. tortilis* seed powder plant extract and MnNPs.

The FTIR spectrum of the *A. tortilis* seed powder extract revealed significant absorption bands at 1,635 cm^−^^1^, indicating N-H vibrations from primary amines (Keshari et al., 2021). An absorption band at 2,156 cm^−^^1^ was assigned to C≡C stretching vibrations of alkyne groups (Al-Bermany et al., 2023), while the band at 3,340 cm^−^^1^ was linked to O-H stretching of carboxylic acid and phenolic groups (Adelodun et al., 2020) as shown in Fig. 4. Similarly, the FTIR spectra of the MnNPs exhibited absorption peaks at 1,635, 2,156, and 3,340 cm^−^^1^.

### Scanning electron microscopy (SEM) and transmission electron microscopy (TEM)

**Figure 5:**
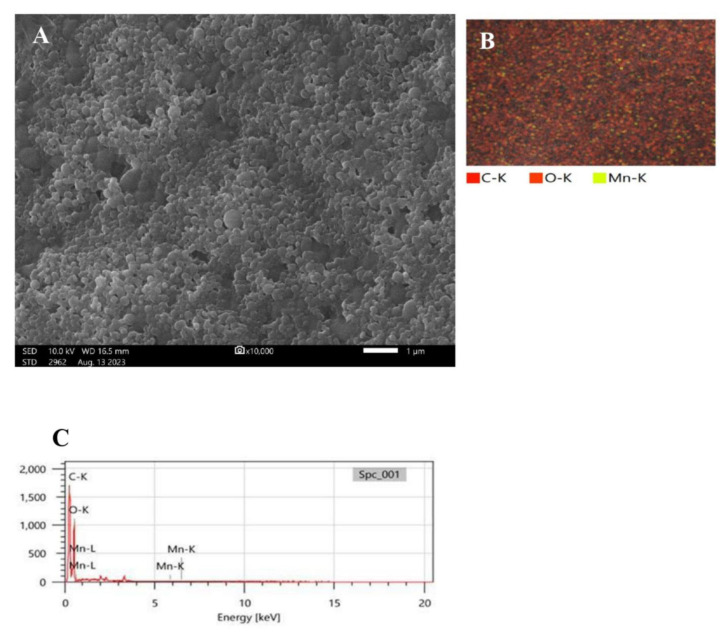
Structural and elemental characterization of MnNPs produced from *Acacia tortilis* seed powder. SEM image for MnNPs produced by *A. tortilis* seed powder (A), their EDX elemental mapping (B) and EDX spectrum (C).

**Figure 6:**
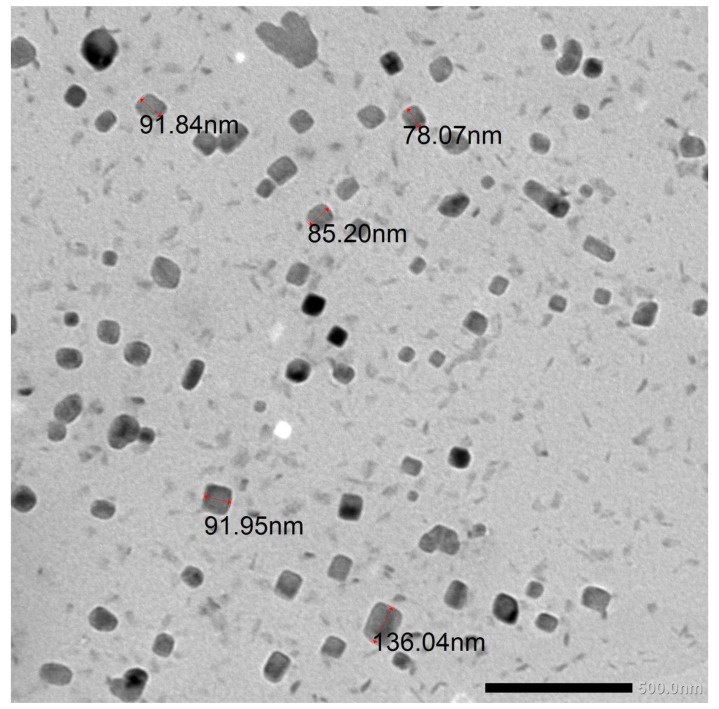
TEM characterization of MnNPs exhibiting mixed cubic and irregular morphologies. TEM image of MnNPs prepared by using seed powder extract of *A. tortilis* representing cubic shape combined with irregular shapes.

The SEM image (Fig. 5A) showed that the nanoparticles predominantly exhibited spherical shapes with some irregular structures. Energy Dispersive X-ray (EDX) analysis revealed a characteristic peak at 0.7 keV, confirming the presence of manganese (Fig. 5B). Elemental mapping (Fig. 5C) demonstrated the distribution of manganese along with carbon and oxygen within the nanoparticles. Transmission Electron Microscopy (TEM) analysis showed that the MnNPs were well-dispersed and mainly exhibited cubic shapes with some irregular morphologies. The average particle size was estimated to be 84.6 nm (Fig. 6).

### Soil analysis and characterization

The analysis and characterization of soil samples from two different locations an industrial area (SI) and a stable site (SS) revealed notable differences in their properties. These variations in soil characteristics are outlined in Table 1, which emphasizes the contrasting environmental conditions of the two locations. Significantly, the organic matter (OM) content is markedly higher in the SS soil. According to the literature, this increased organic matter may compete for active sites, potentially diminishing the effectiveness of MnNPs.

**Table 1:** Analysis and characterisation of soils from an industrial (SI) and a stable area (SS).

Soil samples	SI	SS
pH	8.22	6.61
EC (mmohs/cm)	10.56	15.75
TOC (%)	0.39	0.96
OM (%)	0.67	1.65
Total Nitrogen (%)	0.04	0.13
C/N ratio	9.75	7.38
CaCO_3_(%)	33.8	1.19
Soil texture	Sandy loam	Loamy sand
Sand (%)	58.36	75.41
Silt (%)	34.66	20.11
Clay (%)	6.98	4.48

**Abbreviations:**

EC electrical conductivity TOC total organic carbon OM organic matter C/N ratio carbon to nitrogen ratio CaCO_3_ calcium carbonate

### Potential of MnNPs in tylosin and heavy metals mitigation

**Figure 7:**
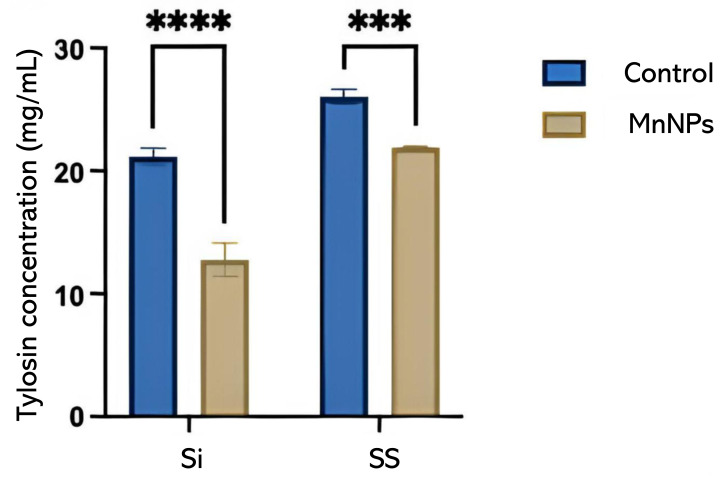
Removal efficiency of tylosin in industrial and stable soils using MnNPs (40 mg/mL, 24 h). Removal efficiency (%) of tylosin from SI and SS soil treated by MnNPs at concentration of 40 mg/mL for 24 h. SI, Soil from an industrial area; SS, soil from a stable area. Data are expressed as mean ± standard deviation (SD) from three independent replicates (*n* = 3). One-way analysis of variance (ANOVA) test was used to assess significance (*p* < 0.05).

**Figure 8:**
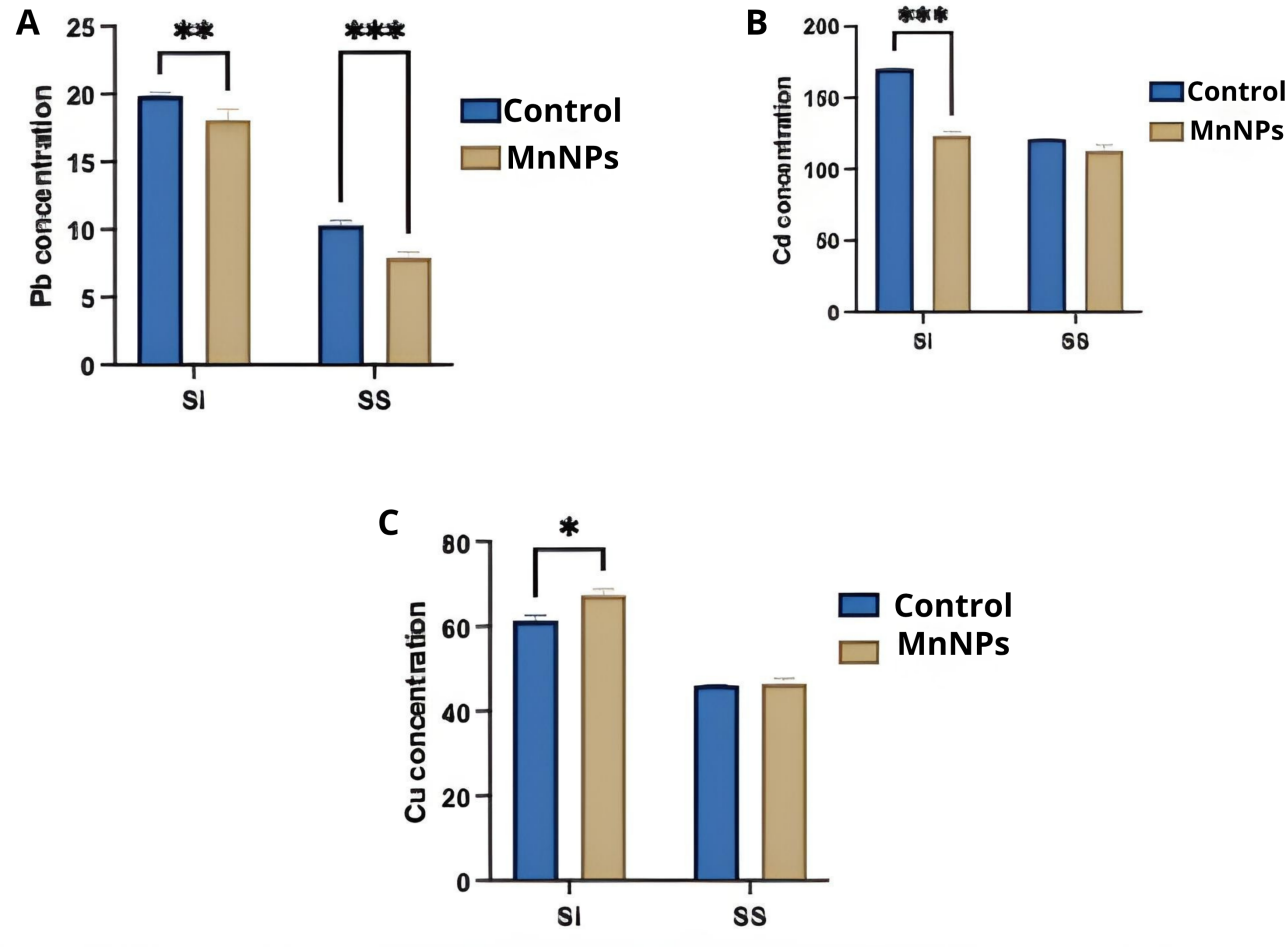
Removal efficiency of Pb, Cd, and Cu from industrial and stable soils using MnNPs (50 mg/mL). Removal efficiency (%) of Pb (A), Cd (B), and Cu (C) from SI and SS soil that treated by MnNPs at concentration of 50 mg/mL. SI, Soil from an industrial area; SS, soil from a stable area. Data are expressed as mean ± standard deviation (SD) from three independent replicates (*n* = 3). One-way ANOVA was used to assess significance (*p* < 0.05).

The application of manganese nanoparticles (MnNPs) reduced tylosin (TYL) concentrations in both soil types (SI and SS), as shown in Fig. 7. Control samples exhibited higher TYL concentrations compared to treated samples. In SI soil, the reduction in TYL concentration reached 39.80%, whereas in SS soil, the reduction was 16.10%. For heavy metals (HMs), treatment with MnNPs resulted in a decrease in metal concentrations (Fig. 8). Lead (Pb) concentrations decreased by 7.46% in SI soil and 22.11% in SS soil (Fig. 8A). Cadmium (Cd) concentrations were reduced by 51.19% in SI soil and 14.90% in SS soil (Fig. 8B). No significant change was observed in copper (Cu) concentrations in either soil type (Fig. 8C).

### Gas chromatography–mass spectroscopy (GC–MS) analysis

The chemical compounds detected in this study from the extract of *Acacia tortilis* seeds were seven key compounds, including cis-Vaccenic acid (C_18_H_34_O_2_), Phenol, 4-ethenyl-2,6-dimethoxy-(C_10_H_12_O_3_), Hexadecanoic acid, methyl ester (C_17_H_34_O_2_), Cyclopentane, 1-pentyl-2-propyl (C_13_H_26_), n-Hexadecanoic acid (C_16_H_32_O_2_), 9-Octadecenoic acid, methyl ester, (E) (C_19_H_36_O_2_), and 9,12-Octadecadienoic acid (Z,Z)-, 2-hydroxy-1-(hydroxymethyl)ethyl ester (C_21_H_38_O_4_). Each compound’s molecular weight, retention time, and match score were recorded to confirm its identity. These data provided the foundation for further *in silico* analyses since these compounds are expected as reducing and capping agents in NPs formulation.

### *In silico*, ecotoxicity and environmental fate assesment of bioactive compounds in *Acacia tortilis* seed extract

The *in-silico* analysis provided a comprehensive evaluation of the ecotoxicity, including earthworm toxicity, and the environmental fate of the seven compounds identified in the extract of *Acacia tortilis* seeds.

### EcoToxicity

**Figure 9:**
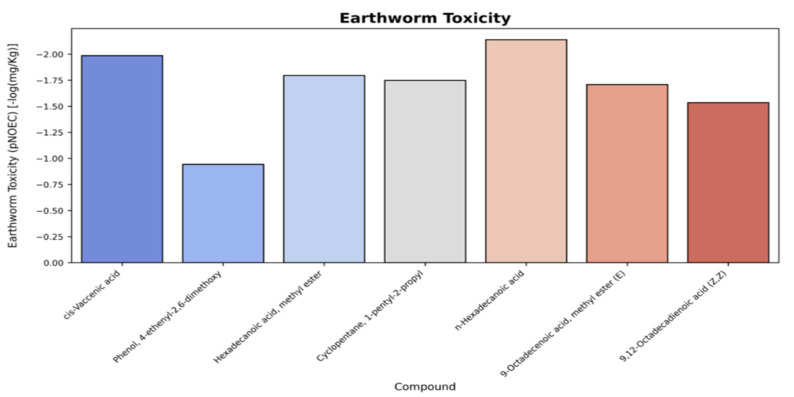
Predicted earthworm toxicity (pNOEC) values for the studied compounds. Earthworm toxicity (pNOEC) values for the analyzed compounds. The pNOEC values are expressed as negative logarithmic concentrations [−log(mg/kg)], with lower values indicating higher toxicity. Predictions were generated using the Earthworm Toxicity Model (CONCERT v.1.0.0), and all compounds fell outside the model’s applicability domain (AD).

The Earthworm Toxicity Model (CONCERT) predicted pNOEC values ranging from −2.138 log(mg/kg) for n-Hexadecanoic acid to −0.943 log(mg/kg) for phenol, 4-ethenyl-2,6-dimethoxy (Fig. 9). The pNOEC values for the other compounds were as follows: cis-vaccenic acid (−1.985 log(mg/kg)), hexadecanoic acid, methyl ester (−1.794 log(mg/kg)), cyclopentane, 1-pentyl-2-propyl (−1.748 log(mg/kg)), 9-Octadecenoic acid, methyl ester (E) (−1.707 log(mg/kg)), and 9,12-Octadecadienoic acid (Z,Z)-, 2-hydroxy-1-(hydroxymethyl)ethyl ester −1.535 log(mg/kg)). Notably, all predictions fell outside the model’s applicability domain (AD).

### Fate and distribution

**Figure 10:**
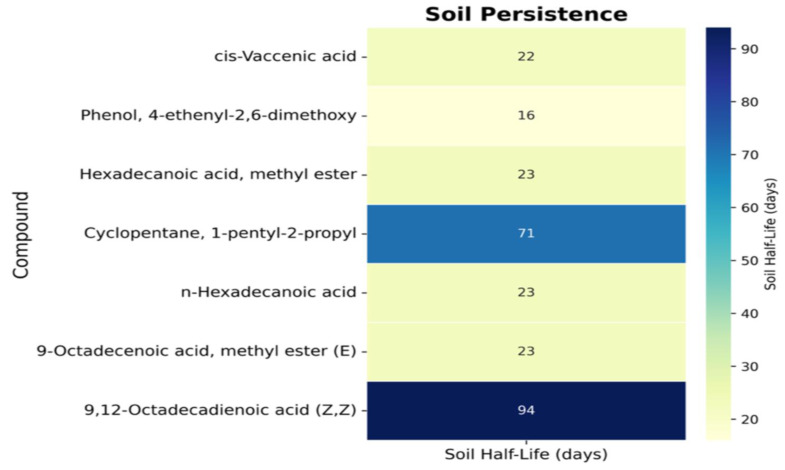
Predicted soil stability (half-life) of the analyzed compounds. Soil persistence (half-life in days) of the analyzed compounds. Compounds with longer half-lives, such as 9,12-Octadecadienoic acid (Z,Z)-, 2-hydroxy-1 (hydroxymethyl)ethyl ester (94 days), indicate higher environmental stability, while compounds like phenol, 4-ethenyl-2,6-dimethoxy (16 days) degrade more rapidly. Predictions were generated using thePersistence (soil) quantitative model (IRFMN v.1.0.1).

The predicted half-life values exhibited significant variation among the compounds, highlighting differences in environmental persistence. As shown in Fig. 10, phenol, 4-ethenyl-2,6-dimethoxy displayed the shortest half-life of 16 days, indicating rapid degradation in soil environments. In contrast, cyclopentane, 1-pentyl-2-propyl and 9,12-Octadecadienoic acid (Z,Z)-, 2-hydroxy-1-(hydroxymethyl)ethyl ester demonstrated the longest half-lives of 71 and 94 days, respectively, suggesting greater environmental stability and potential for prolonged presence. Compounds such as cis-Vaccenic acid, Hexadecanoic acid, methyl ester, n-Hexadecanoic acid, and 9-Octadecenoic acid, methyl ester (E) exhibited intermediate half-lives ranging from 22 to 23 days, indicating moderate persistence.

## Discussion

The study employed *A. tortilis* seed powder as an effective reducing agent for the synthesis of manganese nanoparticles (MnNPs), showcasing an eco-friendly and sustainable approach to nanoparticle fabrication. The resulting MnNPs underwent comprehensive characterization using various analytical and spectroscopic techniques, and their ability to remediate tylosin (TYL) and heavy metals from two types of contaminated soil was assessed. In addition, computational methods were utilized to evaluate the environmental and toxicological properties of key compounds identified from the extract of *Acacia tortilis* seeds, as well as their eco-toxicological impacts and persistence in soil, using *in-silico* models.

The UV-visible spectroscopy analysis of the MnNPs, reveals absorbance peaks in the wavelength range of 200–450 nm, with a prominent peak at 378.32 nm. These findings align with theoretical expectations, as MnNPs generally exhibit absorbance in the 350–410 nm range (Saod et al., 2022). Furthermore, our results are consistent with prior research, such as that by Prasad & Patra (2017), which noted an absorption peak at 360 nm for MnNPs synthesized using *Phyllanthus amarus* extract. These findings confirm the successful synthesis of MnNPs using *A. tortilis* seed powder.

The Dynamic Light Scattering (DLS) technique was utilized to evaluate the hydrodynamic size, polydispersity index (PDI), and surface zeta potential (ZP) of tthe MnNPs exhibited an average hydrodynamic size of 404.1 nm, with a PDI value of 0.14. A PDI below 0.5 indicates a highly monodisperse nanoparticle distribution, reflecting size uniformity (Danaei et al., 2018). Zeta potential analysis indicated an average potential of −32.09 mV for the MnNPs. This negative value suggests significant electrostatic repulsion between the particles, which enhances their stability in suspension (Anandalakshmi, Venugobal & Ramasamy, 2016). In a related study, Asaikkutti et al. (2016), reported the synthesis of manganese oxide nanoparticles using *Ananas comosus* peel extract, which showed a smaller average particle size of 50 nm and a zeta potential of −31.36 mV. This further emphasizes the importance of these characteristics in determining nanoparticle stability and behavior.

*Acacia tortilis* seed powder is rich in secondary metabolites, including flavonoids, alkaloids, saponins, cardiac glycosides, and catechic tannins, all of which play crucial roles in reducing metal salts and serving as capping and stabilizing agents (Snoussi, Najett & Djamel, 2020; Haddi, El Kharraz & Kerroumi, 2024). To examine the changes in bonding that occur during metal reduction and nanoparticle formation, Fourier Transform Infrared (FTIR) spectroscopy was conducted on both the *A. tortilis* seed powder extract and the synthesized manganese nanoparticles (MnNPs). The FTIR spectrum of the *A. tortilis* seed powder extract revealed significant absorption bands at 1,635 cm^−^^1^, indicating N-H vibrations from primary amines. An absorption band at 2,156 cm^−^^1^ was assigned to C≡C stretching vibrations of alkyne groups, while the band at 3,340 cm^−^^1^ was linked to O-H stretching of carboxylic acid and phenolic groups. Similarly, the FTIR spectra of the MnNPs exhibited absorption peaks at 1,635, 2,156, and 3,340 cm^−^^1^. This finding confirms that the MnNPs were effectively capped with secondary metabolites from the *A. tortilis* seed powder extract. A recent study that synthesized manganese dioxide nanoparticles (MnO_2_ NPs) using green tea extract also reported absorption bands at 1,635 and 3,360 cm^−^^1^ (Saod et al., 2022). The presence of diverse functional groups on the surface of the MnNPs may significantly enhance contaminant adsorption (Ahuja et al., 2022).

Scanning electron microscopy (SEM) was used to examine the morphology of manganese nanoparticles (MnNPs). Furthermore, Energy Dispersive X-ray (EDX) analysis was performed to accurately assess the elemental composition of the synthesized materials (Susanti et al., 2023). The spherical morphology observed in SEM images is consistent with previously reported studies, which reported uniformly distributed spherical MnNPs (Khan et al., 2020). The EDX peak at 0.7 keV confirms the successful formation of MnNPs, in agreement with earlier findings (Amatya, Shrestha & Aryal, 2021). The presence of carbon and oxygen in the elemental mapping suggests the involvement of phytochemical compounds from *Acacia tortilis* extract, which likely act as capping and stabilizing agents (Kambale et al., 2020). TEM analysis provided a more accurate estimation of particle size compared to DLS, as it measures the actual physical dimensions rather than the hydrodynamic diameter. The cubic morphology observed in TEM images is comparable to previous studies (Jassal et al., 2016). The differences in morphology between SEM and TEM observations may be attributed to variations in measurement techniques and sampling regions.

Heavy metal contamination in soil poses a significant threat to global food security due to its environmental persistence, bioaccumulation, and physiological disruption (Adnan et al., 2024). Similarly, antibiotic contamination negatively impacts microbial activity, harms non-target organisms, and promotes antibiotic resistance (Mandal, 2024). The World Health Organization has raised concerns about the rising mortality rates associated with antibiotic-resistant infections, highlighting the urgent need for monitoring and mitigation strategies (Nava, Daneshian & Sarma, 2022). Nanotechnology presents a cost-effective remediation option, as it minimizes the transport of contaminated soil and enables the recycling and reuse of nanomaterials (Dhanapal et al., 2024). The observed reduction in tylosin concentrations following MnNPs treatment suggests that nanoparticle-based remediation is effective in reducing antibiotic contamination. This reduction may be attributed to interactions between tylosin molecules and the negatively charged surface of MnNPs, facilitating adsorption processes (Luo et al., 2019). In addition, microbial and enzymatic degradation pathways may contribute to tylosin removal through hydrolysis and oxidation reactions (Wang et al., 2025; Yang et al., 2022).

Similarly, the reduction in heavy metal concentrations indicates the effectiveness of MnNPs in immobilizing metal ions. This process is primarily governed by electrostatic interactions between negatively charged nanoparticle surfaces and positively charged metal ions (Guo, Song & Tian, 2020). However, biological mechanisms such as biosorption, bioaccumulation, and enzymatic transformation may also contribute to metal removal in soil systems (Alabssawy & Hashem, 2024).

The variation in removal efficiency between SI and SS soils can be attributed to differences in soil properties. The lower efficiency observed in SS soil may be related to higher organic matter content, which can compete with contaminants for adsorption sites (Gil-Díaz et al., 2017). This highlights the critical role of soil composition in determining remediation performance.

Manganese nanoparticles offer several advantages for environmental applications due to their relatively low toxicity and their role as essential plant micronutrients (Gonçalves et al., 2022). Their catalytic properties further enhance their suitability for soil remediation (Sobańska et al., 2021).

Various applications of MnNPs for environmental remediation have been explored. For instance, Han, Zhang & Zhao (2017), developed MnNPs mediated by carboxymethyl cellulose (CMC), achieving an 88% efficacy in removing the hormone 17*β*-estradiol from soil. Meanwhile, Wang et al. (2023) created hybrids of reduced graphene oxide and MnNPs (rGO/Mn NPs) using green tea extract, which achieved over 90% removal efficiency for oxytetracycline and tetracycline in wastewater. Additionally, other studies have described MnNPs modified into a hollow mesoporous form to enhance heavy metal adsorption, achieving capacities of 897.0 mg/g for Pb^2^^+^, 437.5 mg/g for Cu^2^^+^, and 354.3 mg/g for Cd^2^^+^.

These modified manganese nanoparticles (MnNPs) demonstrated stability in acidic conditions and effectively treated both laboratory and industrial wastewater, reducing heavy metal leaching in contaminated soils (Zhang et al., 2023). In recent years, conventional *ex situ* remediation methods, such as soil excavation, recapping, surfactant flushing, soil vapor extraction, and thermal desorption, have been widely used (Yang et al., 2024). Meanwhile, innovative *in situ* techniques, including chemical oxidation, sequestration, immobilization, and bioremediation, have garnered attention. Although effective, these methods often face challenges such as significant energy and time demands, high costs, and potential environmental impacts (Qian et al., 2020).

Consequently, manganese nanoparticles could present a favorable alternative. However, further research is needed to assess the large-scale application of green metallic nanoparticles, focusing on their soil stability, interactions with soil microbiota, behavior within environmental systems, and potential modifications. Furthermore, nanomaterials show strong potential for soil remediation, their long-term environmental fate and ecological safety remain important concerns. In soil systems, nanoparticles may undergo aggregation, transformation, or interactions with organic matter and mineral components, which can modify their stability, reactivity, and toxicity (Haydar et al., 2025). Therefore, evaluating nanoparticle persistence and ecological risks is essential for the sustainable application of nanotechnology in soil remediation.

Overall, these findings support hypothesis (i), demonstrating that MnNPs significantly enhance contaminant removal. The observed variation between soil types confirms hypothesis (ii), highlighting the role of soil properties.

The *in-silico* analysis provided a comprehensive evaluation of the ecotoxicity, including earthworm toxicity, and the environmental fate of the seven compounds identified in the extract of *Acacia tortilis* seeds. The predicted pNOEC values indicate variability in the potential toxicity of the analyzed compounds, with n-Hexadecanoic acid showing the highest potential toxicity and phenol, 4-ethenyl-2,6-dimethoxy the lowest. Despite these constraints, the results reveal differing levels of risk to terrestrial organisms, with phenol, 4-ethenyl-2,6-dimethoxy and 9,12-Octadecadienoic acid (Z,Z)-, 2-hydroxy-1-(hydroxymethyl)ethyl ester showing relatively lower earthworm toxicity compared to the other compounds. These findings emphasize the need for experimental validation to corroborate computational predictions.

The variation in predicted half-life values indicates differences in environmental persistence, where compounds with shorter half-lives may degrade more rapidly, while those with longer half-lives may persist in soil environments for extended periods. Earthworm toxicity predictions are particularly relevant from an ecological perspective because earthworms are well-established soil bioindicators and play key roles in nutrient cycling, organic matter decomposition, and soil structure formation (Whig, Banerjee & Achanta, 2026). Compounds with lower predicted no-observed-effect concentrations (pNOEC) could, at sufficiently high environmental levels, significantly affect these biological processes. However, QSAR-based ecotoxicological predictions must be interpreted cautiously, as their reliability depends on whether the compounds analyzed fall within the model’s applicability domain (AD). Predictions for compounds outside the AD are associated with higher uncertainty due to differences between the evaluated chemical and those used to train the model. Consequently, computational ecotoxicity analyses are best considered as screening tools that provide preliminary insights into potential environmental hazards. These predictions should always be complemented with experimental testing to accurately assess ecological impacts (Mora et al., 2024; Fisher et al., 2025).

These findings are consistent with previous research emphasizing the variability in the environmental impact of plant-derived compounds. Studies on fatty acids, including n-Hexadecanoic acid, have highlighted their ecological significance and potential toxicity, supporting the observations presented in this study (Afolayan, Odeyemi & Salaam, 2024). However, discrepancies were observed, particularly regarding the diminished efficiency of computational predictions for compounds falling outside the applicability domain (AD). These results underscore the necessity for experimental validation to enhance and refine *in silico* models. This study demonstrated the utility of *in silico* tools in assessing environmental and toxicological risks. The predictive models offered rapid insights into the ecotoxicological properties and environmental persistence of the analyzed compounds, effectively guiding the evaluation of their remediation potential. Additionally, computational methods proved to be cost-effective and efficient, allowing for the analysis of multiple compounds without extensive laboratory experimentation.

Despite its strengths, the study encountered limitations. Several predictions were made outside the applicability domain (AD), which diminished their reliability. Moreover, computational analyses are inherently limited by the quality and scope of the models used, highlighting the importance of experimental validation. Variations in soil composition and real-world conditions could also affect the behavior of these compounds, aspects that *in silico* models may not fully account for. The findings of this study have significant implications for soil remediation strategies. Compounds with shorter soil half-lives, such as Phenol, 4-ethenyl-2,6-dimethoxy, could be prioritized for immediate applications, while those with longer persistence, such as 9,12-Octadecadienoic acid (Z,Z), require further investigation to address potential accumulation risks. Future research should focus on validating these computational predictions through experimental studies and exploring the synergistic effects of these compounds in complex environmental matrices. Furthermore, enhancing model accuracy by incorporating additional experimental data and refining AD thresholds could improve the reliability of *in silico* analyses.

## Conclusion

This study demonstrates that green-synthesized manganese nanoparticles (MnNPs) effectively reduce tylosin (TYL) and heavy metal (HM) concentrations in co-contaminated soils, with efficiency strongly influenced by soil-specific characteristics. MnNPs were more effective in Si soil than in SS soil, underscoring the need to tailor remediation strategies to individual soil types. These findings highlight the potential of MnNPs as a sustainable soil remediation tool, capable of addressing global challenges such as antibiotic resistance and threats to food security. Despite these promising results, several limitations remain. Variability in soil composition, partial contaminant degradation, and uncertainties regarding the long-term behavior, stability, and accumulation of MnNPs pose challenges for field application. Interactions with soil microorganisms, plants, and other environmental components may further influence remediation efficiency and ecological safety. Therefore, careful consideration of environmental risks, experimental validation, and ongoing monitoring are essential for safe and effective implementation. The study also employed computational methods to evaluate the toxicological properties and environmental fate of compounds extracted from *Acacia tortilis* seeds. GC–MS analysis identified seven major compounds with variable behavior: n-Hexadecanoic acid posed high toxicity risks, whereas phenol, 4-ethenyl-2,6-dimethoxy degraded rapidly with lower toxicity. These results emphasize the value of integrating *in silico* predictions with laboratory experiments, prioritizing compounds with shorter soil half-lives, and refining remediation strategies based on both soil and contaminant characteristics. Overall, MnNPs represent a promising approach for targeted, sustainable soil remediation. Future research should focus on their long-term environmental impact, interactions within soil ecosystems, and performance under diverse field conditions to ensure reliable and safe large-scale application.

## Supplemental Information

10.7717/peerj.21443/supp-1 Data S1 Raw data of the removal efficacy (%) of MnNPs in Si and SS soils for tylosin (TYL) and Heavy metals Table A. Raw concentration data of tylosin (TYL) in Si and SS soils before (control) and after MnNPs treatment, as determined by LC–MS for removal efficiency assessment.
Table B. Raw concentration data of lead (Pb) in Si and SS soils before (control) and after MnNPs treatment, as determined by ICP–Pro for removal efficiency assessment.
Table C. Raw concentration data of cadmium (Cd) in Si and SS soils before (control) and after MnNPs treatment, as determined by ICP–Pro for removal efficiency assessment.

## Additional Information and Declarations

### Data Availability

The following information was supplied regarding data availability: The data is available in the Supplementary Files.
